# Online Peer Support for People With Parkinson Disease: Narrative Synthesis Systematic Review

**DOI:** 10.2196/35425

**Published:** 2022-07-27

**Authors:** Esther Vera Gerritzen, Abigail Rebecca Lee, Orii McDermott, Neil Coulson, Martin Orrell

**Affiliations:** 1 Institute of Mental Health Mental Health and Clinical Neuroscience, School of Medicine University of Nottingham Nottingham United Kingdom; 2 Population and Lifespan Health, School of Medicine University of Nottingham Nottingham United Kingdom

**Keywords:** Parkinson disease, web-based health community, online peer support, narrative synthesis, systematic review

## Abstract

**Background:**

Parkinson disease (PD) significantly impacts the lives of people with the diagnosis and their families. In addition to the physical symptoms, living with PD also has an emotional impact. This can result in withdrawal from social roles, increasing the risk for social isolation and loneliness. Peer support is a way to stay socially connected, share experiences, and learn new coping skills. Peer support can be provided both in person and on the internet. Some of the advantages of online peer support are that it overcomes geographical barriers and provides a form of anonymity; moreover, support can be readily available when needed. However, the psychosocial impact of PD is still underresearched, and there is no systematic synthesis of online peer support for people with PD.

**Objective:**

This review aims to explore the benefits and challenges of online peer support and identify successful elements of online peer support for people with PD.

**Methods:**

The method selected for this systematic review is narrative synthesis. A total of 6 databases were systematically searched in April 2020 for articles published between 1989 and 2020. The quality of the included studies was assessed using the Critical Appraisal Skills Programme qualitative research checklist and the Downs and Black quality checklist.

**Results:**

A total of 10,987 unique articles were identified through a systematic database search. Of these 10,987 articles, 8 (0.07%) were included in this review. Of the 8 studies, 5 (63%) were of good or high quality, 2 (25%) were of medium or fair quality, and 1 (13%) study was of poor quality. Web-based platforms included discussion forums, a web-based virtual world, and Facebook groups. Most papers reported on text-based communication. The included studies reported on sharing social support and personal experiences. Successful elements included increasing similarity between members and offering the opportunity to directly ask questions to a physician. Challenges included members leaving without a warning and PD symptoms hindering the use of technology.

**Conclusions:**

Peer support can improve social support and help people with PD in living meaningful and satisfying lives. Peer support is unique and cannot be replaced by family members, friends, or health care professionals. Online peer support can be a solution for those who do not have access to an in-person support group or whose PD symptoms restrict them from travelling. However, research on the personal experiences of those who engage in online peer support and potential barriers in accessing it remains limited. Future research could use qualitative methods to explore these fields further.

## Introduction

### Background

Parkinson disease (PD) is a chronic and progressive, neurodegenerative condition which is characterized by motor symptoms such as tremor, bradykinesia, and rigidity. In addition to the motor symptoms, many patients experience nonmotor symptoms, such as sleep disturbances, depression, and constipation [1]. It was estimated that in 2016, 6.1 million people had PD globally [2]. People with PD typically start developing symptoms in their 60s; however, it can also occur at a younger age [3]. In 2018, in the United Kingdom, >145,000 people were living with PD, of whom 19,690 were younger than 65 years [4].

PD significantly impacts the lives of people with the diagnosis and their families [5,6]. Owing to its chronic and neurodegenerative nature, people with PD need ongoing care and support [6]. In addition to the physical symptoms associated with PD, receiving the diagnosis and living with the condition also has an emotional impact. This includes anxiety for the future, difficulties in managing the condition in daily life, and the impact on the family [7]. PD can affect people’s social lives and how they are involved in different roles, such as their role within the family, social circles, or at work. Receiving a diagnosis of PD and living with the condition can result in withdrawal from such social roles, increasing the risk of social isolation and loneliness [8].

The psychosocial impact of PD can be discussed within the social health framework [9,10]. In this framework, health is viewed in the social domain and includes three dimensions: (1) being able to fulfill potential and obligations, (2) managing life with some level of independence despite living with a health condition, and (3) being able to participate in social activities and work. When focusing on coping strategies and finding a balance between limitations and one’s abilities, people can successfully adapt to living with a chronic condition and still live meaningful and satisfying lives [10]. Dröes et al [9] discussed how the concept of social health relates to people living with dementia, suggesting that it is possible for people with dementia to still participate in the 3 dimensions of social health and perceive a good quality of life. Within the PD context, Vescovelli et al [11] touch upon the social health framework by emphasizing the importance of social support for the well-being of people with PD. Social support is a term used to describe receiving care and help from others. It is often linked to social connectedness and being part of a social network [12]. In their systematic reviews, Vescovelli et al [11] found that for people with PD, social support is associated with greater social inclusion and opportunities to remain involved with work, supporting people to keep living meaningful and satisfying lives despite their PD. Thus, social support could improve the social health of people with PD [11]. However, despite these findings, Hellqvist et al [8] and Vescovelli et al [11] conclude that the psychosocial impact of PD is still underresearched.

One way in which people can stay socially connected and thus improve their social health is through peer support [13]. Peer support can be defined as the exchange of support between those (also referred to as *peers*) who share a similar health condition or life experience [14,15]. Peers can provide one another with social support; more specifically, there is reciprocity of support, meaning that people can develop a relationship in which they can both receive and provide support. This can increase feelings of empowerment [16,17]. Furthermore, peers can share experiential knowledge, which includes information and perspectives that people have because of their personal experiences of living with a certain condition [17]. These elements are unique to peer support and cannot be provided by health care professionals or others who are not living with PD [14,15].

Peer support can be provided in different ways, including web-based settings. The internet is an important source of health-related information and provides a platform for the creation and spread of web-based patient communities [16]. Since the 1990s, the number of web-based patient communities for a variety of health conditions has been increasing [18,19]. Such communities can function as self-help groups in which members share experiences and emotions and provide mutual support and empathy [16,20,21]. Some of the advantages of online peer support compared with in-person support groups include that it overcomes geographical barriers [19,22]; provides a form of anonymity, which can be particularly suitable for people with stigmatized conditions [22,23]; and online peer support can be readily available at any time of the day when needed [19,22]. Research has been conducted on online peer support communities for a variety of health conditions, including chronic conditions such as multiple sclerosis (MS) [24,25], HIV or AIDS [26,27], and amyotrophic lateral sclerosis (ALS) [28,29]. The review by Kingod et al [13] shows that online peer support communities can offer people with chronic conditions emotional, social, and practical support in managing their condition in their daily lives. Chronic conditions covered by this review include type 1 diabetes, HIV or AIDS, and chronic pain [13].

Web-based health communities and peer support in web-based settings is a rapidly growing field [16,18,19]. Especially during the COVID-19 pandemic and national lockdowns, connecting with others on the web has become increasingly important. However, knowledge of the long-term effects of online peer support, how it impacts users’ health and self-management, and what particular elements make it useful and meaningful need further research [19,30]. Research into online peer support for people affected by PD is also growing [31,32]; however, to the best of our knowledge, there is no systematic synthesis of this research yet.

### Objectives

This narrative synthesis systematic review aimed to (1) explore the benefits and challenges of online peer support and (2) identify successful elements of online peer support for people with PD. In this review, the challenges cover things that make it more difficult for a person with PD to use online peer support. This can include aspects related to technology as well as PD-related challenges. Understanding the successful elements can be helpful in improving existing and developing new online peer support opportunities for people with PD as well as other conditions. Elements of online peer support were deemed successful if studies identified positive outcomes for the people with PD engaging in online peer support.

## Methods

### Narrative Synthesis

The method that was selected for this systematic review was narrative synthesis, using the procedures outlined by Popay et al [33]. This entails including the following elements: (1) theory development, (2) development of a preliminary synthesis, (3) exploration of relationships in the data, and (4) assessment of robustness of the synthesis. With a narrative synthesis, the presentation of the findings is mainly words- and text-based, and it is a useful method to identify elements of best practice [33]. Furthermore, this review followed the PRISMA (Preferred Reporting Items for Systematic Reviews and Meta-Analyses) 2020 guidelines [34]. More details on the narrative synthesis methods can be found in Multimedia Appendix 1 [9,10,14,16,33,35].

### Search Strategy

A systematic database search was conducted in April 2020. The search strategy was developed with the help of 2 librarians and NC, who is an academic expert on online peer support. The initial search was part of a wider appraisal of the literature and included PD, MS, ALS, and Huntington disease. This paper will only present the results for patients with PD. A total of 6 databases were searched: CINAHL, Cochrane Library, EMBASE MEDLINE, PsycINFO, Scopus, and Web of Science. The keywords used for the searches are presented in Textbox 1. A search filter for the year of publication, 1989 to 2020, was applied. This was because the World Wide Web was introduced in 1989. No filters on the study design were applied. Finally, the reference lists of the included papers were searched manually. This did not result in any new papers being added.

**Search terms.**

**Search term 1**

- parkinson* disease
- parkinson*

**Search term 2**

- online
- digital
- web-based
- app-based
- internet
- social media
- peer
- peer support
- support group
- social support
- online support group
- online support commun*
- discussion forum*
- bulletin board
- chat room*
- computer-mediated support
- internet support group*
- internet support commun*
- online self-help
- web-based support group*
- web-based support commun*

Textbox 2 lists the inclusion and criteria followed while selecting papers for this review.

**Inclusion and exclusion criteria.**

**Inclusion criteria**

- The study population included people living with Parkinson disease or a blend of people living with Parkinson disease and caregivers.
- The intervention included online peer support. For this review, online peer support was regarded as communication via the internet between peers in a web- or app-based environment that is designed to facilitate social contact using either an asynchronous or synchronous text- or text and video-based platform (eg, social media platforms, forums, or chat rooms).
- Publication between 1989 and 2020.
- Publication in peer-reviewed journals.

**Exclusion criteria**

- The study focused solely on caregiver perspectives.
- The intervention included online peer support that was part of a program that also included in-person or telephone-based peer support.
- The study did not report on peer-to-peer interactions. This exclusion criterion was added after initial screening. See the *Study Selection* section for more details.
- Literature reviews, opinion pieces, protocols, editorials, or conference abstracts.
- Papers written in a language other than English if a translation was not available.

### Study Selection

The search results were imported into EndNote (Clarivate), after which all duplicates were removed. The primary reviewer (EVG) reviewed each title and abstract against the eligibility criteria. The primary reviewer consulted a second reviewer (ARL) on the titles and abstracts that she was unsure about. The title and abstract screening was followed by a full-text analysis of the potentially relevant papers. The initial full-text analysis was conducted by the primary reviewer. The same procedures as used for the title and abstract screening were followed. At this stage, the main reason for labeling a paper as unsure was that although the paper met the inclusion and exclusion criteria, it mainly focused on other outcomes (eg, quality of life) rather than peer-to-peer interactions. Following a discussion with a third reviewer (OM), it was decided to refine the exclusion criteria and add the criterion that papers could be excluded if they did not report on peer-to-peer interactions. The papers that were included up to that point were reassessed against the newly added exclusion criterion.

### Data Extraction

Following the study selection, the primary reviewer (EVG) extracted the data using standardized data extraction forms. Data were extracted on (1) study information, (2) study characteristics, (3) population characteristics, (4) characteristics of the web- or app-based platform, (5) outcomes, and (6) implications for future research. ARL provided a second independent review of the completed data extraction forms.

### Quality Assessment

In all, 2 quality assessment tools were used to assess the risk of bias in individual studies. EVG completed the initial quality assessment and ARL provided a second independent review. For the assessment of the risk of bias in qualitative studies, the Critical Appraisal Skills Programme (CASP) qualitative research checklist was used [36]. This checklist consists of 10 questions related to “rigour, credibility and relevance” [37]. For studies that could not be assessed using the CASP checklist, the Downs and Black quality checklist was used. This tool consists of 27 items and is suitable for both randomized and nonrandomized studies [38]. Both the CASP checklist and the Downs and Black quality checklist were recommended by the Centre for Reviews and Dissemination guidance for undertaking reviews in health care [37] and have been successfully used in previous systematic reviews [39,40].

For the CASP checklist, studies will be graded *high*, if they met or partially met 8 to 10 items; *medium*, if they met or partially met 5 to 7 items; and *low*, if they met or partially met 0 to 4 items [41]. For the Downs and Black quality checklist, papers are labeled *excellent*, if they have 24 to 28 points; *good*, if they have 19 to 23 points; *fair*, with 14 to 18 points; and *poor*, when they have less than 14 points [42].

## Results

### Overview

**Figure 1:**
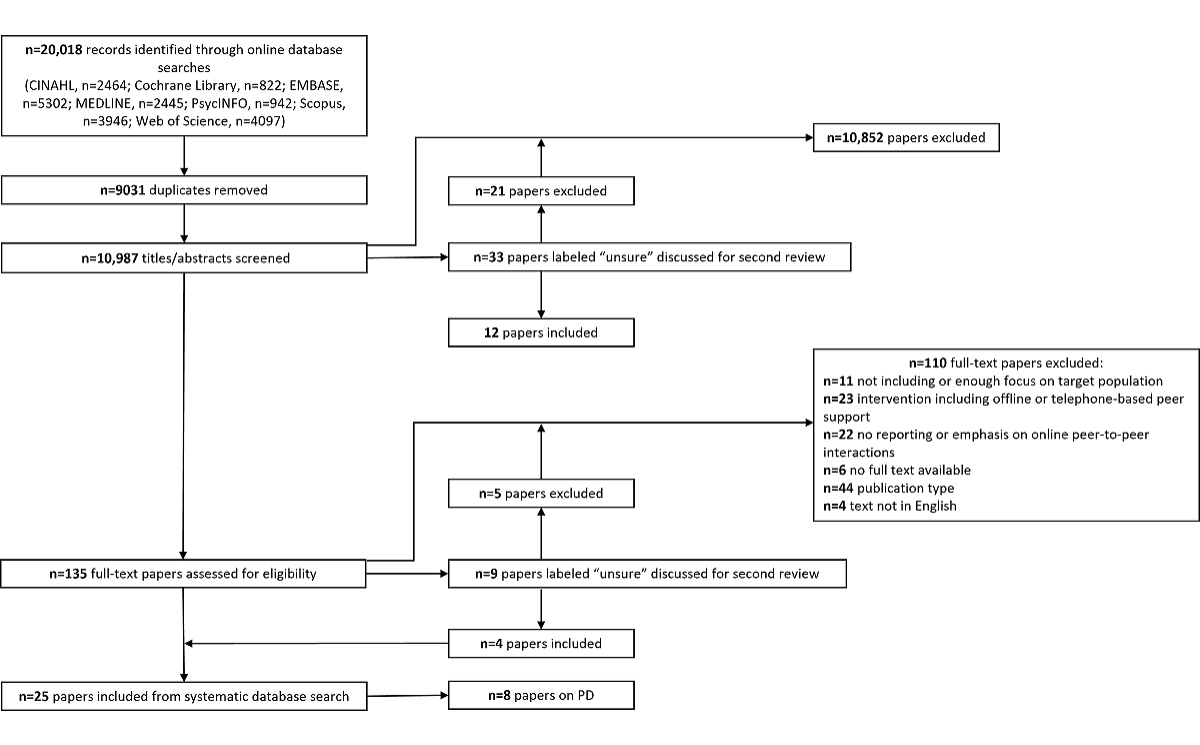
PRISMA (Preferred Reporting Items for Systematic Reviews and Meta-Analyses) diagram of the search and review process. PD: Parkinson disease.

The results section covers element 2 of a narrative synthesis: developing a preliminary synthesis. A web-based database search returned 10,987 unique titles and abstracts. After screening of the titles, abstracts, and full texts, of the 10,987 papers, 8 (0.07%) met the inclusion criteria for this review. An overview of the web-based database search and screening process can be found in Figure 1.

### Study Characteristics

An overview of the study characteristics is presented in Table 1. This review includes a variety of methods. Of the 8 papers, 3 (38%) papers used a qualitative content analysis of posts on a discussion forum [31,32,43], 3 (38%) papers reported the findings of a pilot study [44-46], 1 (13%) paper conducted an ethnographic study in a virtual world [47], and 1 (13%) paper conducted a survey and interviews [48].

**Table 1:** Study characteristics.

Study (year)	Aim or aims		Design (methods)	Intervention	Setting (country)		Study population				Eligibility criteria	Sample			QA^a^ score
Attard and Coulson [31] (2012)	Experiences of PD^b^ forum users		Qualitative content analysis of posts on 4 discussion forums	On the internet, public, asynchronous discussion forum	Study conducted in the United Kingdom; data collected from the United States, Canada, and Australia		People living with PD				PD online support groups with a discussion forum	A total of 4 web-based communities; 1000 to 10,000 members per group; approximately 100 active members per group; age unknown (only what members decided to share); more women than men; 1013 messages (approximately 250 per group)			9 (high)
Bakke et al [32] (2018)	Interaction between professional and personal expertise in web-based PD community		Qualitative content analysis of posts on discussion forum	On the internet, public, asynchronous discussion forum	Unknown		People living with PD and carers				Physician-moderated forum for PD	In all, 1 web-based community: 107 threads, 409 individual comments; age and gender unknown (only what members decided to share)			8 (high)
Stewart Loane et al [43] (2014)	Social support and consumer value in web-based health communities		Qualitative content analysis of posts on discussion forum	On the internet, asynchronous discussion forum	Unknown		People with PD				Not reported	PD community: 35 members, 30 threads, 137 posts; age and gender not reported			8 (high)
Davis and Boellstorff [47] (2016)	Creativity of people with PD in a virtual world		Qualitative ethnographic web-based study in a virtual world	Second Life, a virtual world	Study conducted in the United States (based on ethics approval)		People living with PD				Members of a PD community in a virtual world (recruited through prior fieldwork in 2004)	A total of 2 people living with PD (1 male and 1 female); female patient with young onset PD, male patient with unknown onset			7 (medium)
Lieberman et al [46] (2005)	Impact of group composition and utility of computer-based text analysis in developing web-based groups		Pre-post measurement study comparing homogeneous and heterogeneous groups	A total of 6 web-based PD support groups delivered by professionals; weekly meetings for 20 weeks; 3 homogeneous groups (2 young onset, aged <60 years; 1 newly diagnosed in the last 2 years); 3 heterogeneous groups (mix of age and time since diagnosis)	Study conducted in the United States		People living with PD				People living with PD in California and attending web-based PD support groups, described in the study by Lieberman et al [46]	A total of 66 participants: 12 were unable to attend, 12 dropouts from homogenous groups, and 9 dropouts from heterogeneous groups; homogeneous groups: mean age 55.6 (SD 6.4) years, 77.8% female; heterogeneous groups: mean age 63.9 (SD 8.5) years, 46.2% female			16 (good)
Lieberman et al [45] (2006) (same population [46])	Willingness to participate in professionally led web-based groups; characteristics of participants; outcomes; group composition		Pilot study of effectiveness of professionally led web-based PD support groups	See above for Lieberman et al [46]	Study conducted in the United States		People living with PD				People living with PD in California	A total of 66 participants: 32 completed pre-post measurements; mean age 60.2 (SD 9.2) years, 68% male			16 (good)
Lieberman [44] (2007; same population [46])	Characteristics of people with PD in online support groups and impact of fear on dropout rates		Pilot study	See above for Lieberman et al [46]; weekly meetings, 90 min per meeting, 25 weeks; premature termination: attending <10 meetings	Study conducted in the United States		People living with PD		People living with PD			A total of 66 participants: 26 premature terminators and 40 continuers			15 (fair)
Martínez-Pérez et al [48] (2014)	Characteristics of Facebook groups and Twitter and their purposes and functions	Mixed methods survey and interviews with Facebook and Twitter users		Facebook and Twitter groups for PD		Unknown		People affected by PD		Facebook and Twitter focused on prevention, treatment, fund raising, cures, or general information			A total of 257 Facebook groups and 100 Twitter groups; no demographic information about group members was presented	4 (low)	

^a^QA: quality assessment.

^b^PD: Parkinson disease.

### Summary of Interventions

In all studies, the mode of communication between the participants was text-based. In 50% (4/8) of studies, communication was asynchronous [31,32,43,48], meaning that participants did not necessarily communicate with each other in real time. This is one of the characteristics of discussion forums, where people can post a message and others can respond at a time that is convenient for them. A total of 50% (4/8) of studies [44-47] used real-time communication (synchronous). Other than in a study [48], all online peer support communities analyzed in this review were moderated. This means that one or multiple people either guided the discussion or monitored posts. Although 75% (6/8) of studies only included people living with a PD diagnosis [31,43-47], 25% (2/8) of studies included both caregivers and people with a PD diagnosis [32,48].

### Quality Assessment

Of the 8 papers, 5 (63%) were of good or high quality, 2 (25%) were labeled medium or fair quality, and 1 (13%) paper was labeled as poor quality. In total, 63% (5/8) of papers were assessed using the CASP checklist. Of these5 papers, 3 (60%) were labeled as high quality [31,32,43], 1 (20%) as medium [47], and 1 (20%) was assessed to be of low quality [48]. The 38% (3/8) of remaining papers were assessed using the Downs and Black quality checklist. Of these 3 papers, 2 (67%) were labeled as good [45,46] and 1 (33%) was labeled as fair [44]. An overview of the CASP checklist, Downs and Black quality checklist, and the scores for each study can be found in Multimedia Appendix 2 [31,32,43-48].

### Key Findings

#### Overview

An overview of the web-based platform characteristics is presented in Table 2. An overview of the study outcomes is presented in Table 3.

**Table 2:** Web-based platform characteristics.

Study	Platform	Communication	Moderation
Attard and Coulson [31]	Discussion forums	Text-based (asynchronous)	Yes
Bakke et al [32]	WebMD (discussion forum)	Text-based (asynchronous)	Physician
Loane et al [43]	Discussion forum	Text-based (asynchronous)	Unknown
Davis and Boellstorff [47]	Virtual world	Verbal (synchronous)	Researchers
Lieberman et al [46]	Online support group in chat room	Text-based (synchronous)	Professional
Lieberman et al [45]	Online support group in chat room	Text-based (synchronous)	Professional
Lieberman [44]	Online support group in chat room	Text-based (synchronous)	Professional
Martínez-Pérez et al [48]	Facebook and Twitter	Text-based (asynchronous)	Unknown

**Table 3:** Study outcomes.

Study	Reported outcomes	Successful elements	Implications
Attard and Coulson [31]	Positives: Social support, mutual understanding, and empathy Sharing experiences and advice Being part of a community, feeling less alone, and friendship Encouragement, positive thinking, and resilience Negatives: Lack of replies Symptoms restricting ability to use computer Lack of personal information Absence of nonverbal communication Members leaving could be distressing for other members	Variety in experience, opinions, and advice Tailored advice to individual members in simple, nonmedical language Writing may help people to reflect on their situation and share things that are difficult to express face to face Anonymous nature may help members to discuss taboo topics more openly	Explore the use of voice tools for people with PD^a^ who have difficulties typing because of their symptoms Ask users directly about experiences Evaluate: accuracy of shared information impact of public nature of forum on members’ experience and concerns about privacy impact of the presence of professional moderators
Bakke [32]	Role of professional expertise: Trust in physician’s opinion Acknowledging value of lived experience Role of lay expertise: Value and trust peer’s experiences. Mutual understanding and empathy Sharing personal experiences Reciprocity in answering questions and info sharing Referring to physician for advice Trust increased over time as members shared more	Having a physician moderator Opportunity to directly ask questions to physician Physician using understanding and supportive tone Peer interaction, receiving advice from others going through something similar Forum design: clearly labeling posts and profiles of physicians may play a role in building trust	For designing future forums: include badges and ratings to add validity to forum users’ contributions clear norms and values pinned to home page Moderation (professional or nonprofessional)
Stewart Loane et al [43]	Information support most frequent, emotional support second. Initial posts often request information. Responses include answers and network and emotional support When sharing info, the posters receive positive feedback Spiritual support (expression of gratitude and feelings of connectedness) Ethics and morality: participants refusing to provide a diagnosis or medical advice Sharing poems and photos, humor, and banter. Sense of community	People with PD developed value through discussion without needing health care professionals to be present. This is helpful for health care professionals and managers. Web-based discussion forums can remove barriers of information asymmetry and they create value and support for people with PD.	Using different methods to directly explore members’ experiences Further explore what features of a web-based community promote a sense of community among members Explore a variety of web-based communities to identify whether specific features lead to greater value for members
Davis and Boellstorff [47]	Users: discovered new ways of creativity continued creative parts of previous jobs which gave sense of purpose created art works in the platform to express what it feels like to have PD felt part of a community beyond PD learned new web-based skills	The Second Life platform was used for offline work purposes Art works created in Second Life to express how it feels to have PD can be used for educational purposes It can be difficult to find age-appropriate in-person support groups for younger people with PD. Web-based platforms are accessible to people from different areas	Explore the influence of factors such as gender, age, and young onset or late-onset PD on creativity Explore to what extent creativity is experienced as a community or an individual phenomenon
Lieberman et al [46]	Quality of life of all groups improved Homogeneous groups: were more committed to their group had higher levels of commitment and attraction, and positive feelings in initial 5 meetings had significantly greater positive changes compared with heterogeneous groups	Homogeneous groups based on age or time since diagnosis The internet makes it easier to create homogeneous groups, with access to a larger group of patients Lurking (reading posts but not creating own posts) can help with learning more about the group and finding similarities with other members	Explore: the impact of writing in online peer support groups the impact of the absence of visual and auditory cues Internet support groups could target a more specific audience to enhance similarity between members Option for subgroups
Lieberman et al [45]	Members of web-based groups: had lower average age were living with diagnosis for fewer years had better scores for depression and QoL^b^ before and after the intervention felt freer to talk about certain topics compared with in-person groups Only homogeneous groups continued to stay in touch after intervention Most participants heard about the online support groups through the internet, only a small percentage through their physician.	Homogeneous groups based on age or time since diagnosis	Explore why people drop out of online support groups Explore opportunities of using voice recognition software
Lieberman [44]	Participants who dropped out: had higher levels of anxiety did not score differently on depression, quality of life, and intensity of PD symptoms measurements	Homogeneous groups showed significantly greater improvement compared with heterogeneous groups	Explore what effective strategies are to prevent people from dropping out (eg, group structure, group composition, and preparation)
Martínez-Pérez et al [48]	On Facebook, the majority was self-help groups On Twitter, the goals of people were to share information and create awareness There is a need for dedicated networking sites for peer support	N/A^c^	Directly explore the experiences of users

^a^PD: Parkinson disease.

^b^QoL: quality of life.

^c^N/A: not applicable.

#### Social Support

One of the main characteristics of online and in-person peer support is social support [14,16]. This finding also came forward in this review, and studies reported on different elements of social support. Through content analysis of discussion forums, studies [31,32,43] observed mutual understanding and empathy among the members of the forum and an exchange of different types of support. This was observed through members sharing personal experiences and both providing and receiving support. The most frequently observed types of support were emotional and informational support. Examples of emotional support and expressions of understanding and empathy from the work of Bakke [32] are as follows:

Hi, I feel your fear and confusion.

[...] I am responding to you mainly because I wanted to tell you that you are NOT alone with your medication problems.

An example of informational support was provided in the work of Stewart Loane et al [43]. A person asked the following:

Does anyone ever experience freezing that lasts for hours on end? Please reply urgently.

Another member responded quickly, and the person who asked the question replied as follows:

[...] I tried several of the methods that you suggested and I have found one that works for me. I’m telling you it WORKS. I’m so excited! I have been so worried about what would happen if I were alone and I froze, and now I have a new freedom. Thank you.

Stewart Loane et al [43] observed that new posts on the forum often started with a request for information and that in their responses, other members shared information, personal experiences, and emotional support. Overall, the authors of all 3 papers observed a real sense of community, belonging, and friendship on each of the platforms, which can be described as network support [43]. An example that illustrates this type of support was seen in the work of Attard and Coulson [31]: “I am glad I found this forum, makes me feel like I am not alone.”

In the study by Lieberman et al [46], the authors researched the impact of group composition. Participants were divided into homogeneous (based on age or time since diagnosis) and heterogeneous groups. Although all groups improved on quality of life scores, participants in the homogeneous groups showed significant improvement in depression and PD symptoms compared with heterogeneous groups. These findings suggest that similarities between group members can improve the outcomes of peer support [46].

#### Benefits of Online Peer Support

Davis and Boellstorff [47] observed how 2 people with PD used the Second Life web-based platform. Through their ethnographic study they found that both participants were able to express themselves creatively on the platform. Through their web-based artworks and creative expressions, both people with PD were able to continue with creative parts of their previous jobs, and they also used art to express what it feels like to have PD. A sense of community was also observed here. Furthermore, one of the participants was living in a rural area, where it was difficult to find in-person support groups. In this case, the web-based platform provided a way to connect with other people with PD [47]. The work of Lieberman et al [45] showed that people with PD who participated in web-based groups felt freer to talk about certain topics compared with in-person groups. A participant shared the following [45]:

In an internet group, you are much freer to talk about things that you probably wouldn’t in a F2F [face to face]. We got into discussion of sex [meds affecting sexual desire]. I know I wouldn’t have discussed in a F2F.

#### Challenges of Online Peer Support

Of the 4 studies, only 1 (25%) reported on the challenges related to online peer support communities for people with PD—a qualitative content analysis of a PD discussion forum [31]. Challenges were related to online peer support and the use of technology in general. Some were related to the behavior of group members, such as a lack of replies to posts and group members leaving without warning. This could be distressing for other members. An example that illustrates this is, “If you are out there please respond. I have searched the net for you dear friend and I would like to talk to you again” [31]. Other challenges were more related to the nature of discussion forums and web-based support in general, such as the absence of nonverbal communication, which at times could lead to misunderstandings, and the lack of personal information. Finally, some posts showed that, at times, it was difficult for people with PD to use a computer or other types of technology because of their symptoms: “Sometimes my PD prevents my fingers from being able to type. At other times they work fine, but my brain is a blob!” [31].

Furthermore, a study investigated the reasons why people would drop out of online PD support groups. Findings show that people who dropped out of the online peer support sessions had similar scores on depression, quality of life, and PD symptoms scales but had higher levels of anxiety before starting their participation [44].

#### Successful Elements of Online Peer Support

Several successful elements of online peer support for people with PD have been identified in this review. First, writing may help people reflect on their own situation and share things that may be difficult to express face to face [31]. Second, having homogeneous groups based on age or time since diagnosis leads to increased benefits for members [44-46]. The findings of Lieberman et al [46] show that people who participated in the homogeneous groups felt more committed to their group and had more positive feelings about the group during the first 5 meetings. Furthermore, only members from the homogeneous groups continued to stay in touch after the intervention ended [45]. Finally, although most studies included in this review analyzed moderated platforms, the study by Bakke [32] specifically looked at a physician-moderated platform. The author observed that members appreciated the opportunity to ask questions directly to a professional. A helpful feature in the forum design was clearly labeling the physician’s comments [32].

## Discussion

### Principal Findings

This section presents the summary and interpretation of the findings, covering narrative synthesis element 3: exploring relationships within and between studies. To the best of our knowledge, this is the first review to systematically synthesize the literature on online peer support for people with PD. This review shows that online peer support can be a way for people with PD to stay socially connected, share experiences, and exchange support for managing daily life with PD. Furthermore, this review identified the successful elements of online peer support.

### Benefits and Successful Elements of Online Peer Support

#### Overview

The main positive elements related to peer support are reciprocity and social support [14,16]. This finding has also been identified in this review, indicating that the benefits of peer support are not limited to in-person settings. Despite not knowing each other in person and not being physically close, this review shows that people with PD can find emotional support, mutual understanding, and empathy through web-based communities. Moreover, people with PD can build new friendships and expand their social networks. People can share their personal experiences and provide and receive informational support and advice from others in similar situations. For example, people can share experiences with medication or how they manage PD symptoms in daily life. This is based on experiential knowledge, which is a combination of unique knowledge and expertise that people have because of their personal experiences of living with PD [17]. Sharing knowledge and learning from others’ experience can contribute to developing coping skills for living with PD. This, in turn, can support people in living meaningful and satisfying lives despite having PD [10]. Similar findings have been published on online peer support groups for other conditions, including people with chronic illnesses [13] and Huntington disease [49,50]. This review supports previous research in that the benefits of peer support are not limited to a physical, in-person setting but can also be transferred via the internet. Elements that can make online peer support successful include having homogeneous groups [44-46] and having the option for participants to directly ask questions to a physician [32]. However, different people have different needs and preferences. Some who engage in online support may still miss in-person human interactions such as having a cup of tea together or being able to give someone a hug when they are upset [51].

There are also additional benefits to peer support in a web- or app-based setting. First, online peer support groups are available to a wide range of people, including those living in remote areas. For these people, it might be difficult to find in-person peer support groups in their local areas. PD symptoms may also impose additional challenges on people to travel to in-person peer support groups. Finally, the internet provides a form of anonymity. The anonymous nature of online peer support groups can make it easier for people to discuss taboo topics that are difficult to talk about in an in-person setting [23,45].

#### Challenges of Online Peer Support

Only a few studies in this review provided information on users’ age or gender [44,46,47], whereas for the other studies, it was unknown. Information on group composition and personal information, such as age, gender, or time since diagnosis, is often unknown. A lack of such information can make it difficult to determine the extent to which members have things in common. This also highlights the challenge for people with PD in finding more specific peer support groups, such as young onset PD groups or groups for people who are newly diagnosed. The importance of similarity between group members was presented in the work of Lieberman et al [46]. These findings highlight a key element of peer support and something that defines whether someone is a peer: sharing similarities [14]. A lack of personal information was mostly the case for papers analyzing discussion forums, which could be because of the anonymous nature of such forums. The studies in this review that analyzed a discussion forum all used a publicly accessible platform. Reasons for using publicly accessible forums include ethical issues regarding informed consent and respect for members’ privacy [31]. It could be that because of the public nature, either members did not have the option to share more personal information or members chose not to share that information [19].

#### Impact of Research Methods

Qualitative content analysis was conducted in 38% (3/8) of papers included in this review. Although this method provides insights into what is happening and being shared on the platform, it does not provide information about members’ personal experiences. A number of aspects of this methodology remain unknown. First, the findings are highly dependent on researchers’ interpretations. Although researchers can interpret the intention or underlying meaning of a post, it is often not possible to directly contact the author of the post and ask if this was indeed how they intended their message. Similarly, it is often not possible to directly contact the intended receiver of the post to confirm if they perceived the message in the way that the researcher interpreted it. These challenges can be addressed using qualitative research methods to directly explore users’ experiences, as was done by Davis and Boellstorff [47] and Martínez-Pérez et al [48], or by setting up an online peer support intervention and performing pre-post measurements, as was done by Lieberman et al [45]. Second, on discussion forums and social media pages, all group members can often read all posts (besides private messages). This means that not only the intended receiver but also other members can read the posts. Many people can read it, but not everyone will respond to or participate in the discussion. When using a content analysis method, it remains unknown how people who only read the posts but not interact, also called *lurkers*, interpret the message and experience it [23]. Steadman and Pretorius [52] explored the impact of a Facebook group for people with MS on nonactive members. During individual interviews, people expressed that they still experienced social support despite not being actively involved in the discussions [52].

Third, the research into online peer support presented in this review might show an overly positive image of the online peer support group, as people who are active on the platform and post messages are often the ones that enjoy being part of the community. In many web-based communities, people can come and go when they want, and those who have negative experiences can leave the group without giving a reason. This means that negative experiences and potentially harmful aspects of online peer support groups remain underresearched. A potential negative experience identified in this review is the lack of response to messages [31], which has also been identified in another systematic review [23]. The authors stated that new members of an online peer support group are especially at risk of withdrawing after not receiving a response to their messages. The reason for this could be that new members may be more psychologically vulnerable and have certain expectations when joining the online peer support group [23]. When selecting a specific platform for research on online peer support, there is a risk of presenting an overly positive view of the platform and the experiences of its users. An alternative could be to explore the experiences with and opinions on online peer support in the wider PD community, for example, through a survey.

### Limitations

This section covers narrative synthesis element 4: assessing the robustness of the synthesis. This systematic review only included studies on written communication between people with PD on publicly available platforms. The database search did not identify any papers that included other platforms that can potentially be used for online peer support, such as videoconferencing platforms or social media platforms such as WhatsApp or Instagram. Therefore, the findings of this review are limited to the platforms covered in this review (discussion forums and Facebook groups) and cannot be generalized beyond these. Moreover, of the 8 studies, only 1 (13%) study has included findings on the potential challenges of online peer support [31]. As a result, this review may overrepresent the positive and beneficial aspects of online peer support and may not provide an accurate picture of the real-world experiences of people with PD who are part of such communities. In addition, within the studies, it was sometimes difficult to identify the contributions of technological, social, and individual elements to how people experienced online peer support. Finally, people have different preferences and needs, and online peer support may not be suitable for everyone living with PD. In addition, the physical symptoms of PD may be a barrier for people to use technology and to access online peer support communities. The views and experiences of people who are unable or do not want to engage in online peer support groups have not been presented in this review.

### Recommendations for Future Research

For this review, no papers were identified that covered videoconferencing platforms that can be used for peer support; for example, Zoom, Skype, or Microsoft Teams. As these platforms have become more widely used since the COVID-19 pandemic, future research could explore how widely they are used among people with PD, and if and how they are used for peer support. Furthermore, research could focus on how people experience this form of online peer support and how it impacts their lives, as it is different in nature than what has been discussed in this review. More specifically, videoconferencing platforms include synchronous and verbal communication, often where one sees the other members. This reduces anonymity and adds a face-to-face element, in which nonverbal communication can be more prevalent.

Future research could also focus on using different methodologies for analyzing online peer support for people with PD. Direct assessment of users’ personal experiences was also recommended by some of the studies included in this review [31,43,48]. Examples of these methods include individual interviews, focus groups, or surveys. It is necessary to learn how people with PD truly experience being part of an online peer support community and what the impact is on their daily lives. Furthermore, future research is needed to explore potential negative experiences people may have with online peer support, as these are currently underresearched. Qualitative methods, such as individual interviews and open-question surveys, can be used for this purpose. In addition, there is a group of people who are unable to access online peer support or use technology, for example, because of their PD symptoms. It is important to explore in more detail the barriers that people face and how they could overcome them. Some of the studies included in this review recommended investigating the use of voice assistive tools for people with PD [31,45]. Research into the use of such assistive tools for online peer support has already been conducted for people with ALS; for example, in the work of Caron and Light [53].

### Conclusions

Peer support can be an extremely valuable source of social support for people with PD. More specifically, peer support can improve social health and support people with PD in living meaningful and satisfying lives, despite their condition. Sharing experiences with peers can improve feelings of empowerment and social connectedness and help people with PD develop new coping skills. Peer support is unique and cannot be replaced by family members, friends, or health care professionals who do not live with PD. The benefits of peer support are not limited to physical, in-person support groups but can be transferred via the internet. Online peer support is accessible to a wide range of people and is not limited by geographical barriers. This could make online peer support particularly suitable for those who do not have an in-person peer support group in their local area, or whose PD symptoms hinder them from traveling. However, research on the personal experiences of those who engage in online peer support and potential barriers to accessing online peer support remains limited. Future research could use qualitative methods, such as individual interviews, focus groups, and open-question surveys to explore these fields further.

## Appendix

Multimedia Appendix 1 Narrative synthesis methods.

Multimedia Appendix 2 Quality assessment tables.

## Abbreviations

ALS: amyotrophic lateral sclerosis
CASP: Critical Appraisal Skills Programme
MS: multiple sclerosis
PD: Parkinson disease
PRISMA: Preferred Reporting Items for Systematic Reviews and Meta-Analyses
