# Supplementary material for: Online Peer Support for People With Parkinson Disease: Narrative Synthesis Systematic Review
Source: JMIR Aging. 2022 Jul 27;5(3):e35425. doi: 10.2196/35425 (PMC9377481; doi:10.2196/35425)
Supplement: Multimedia Appendix 1 [file aging_v5i3e35425_app1.docx]

## Multimedia Appendix 1

The aims of Element 1: Theory development are to inform the research questions and the type of studies to include, to support the interpretation of the findings, and to assess the potential generalizability of the findings [33]. One of the key elements of peer support is social support [14, 16]. Research demonstrates that supportive social relationships can promote health and overall wellbeing. Receiving social support and believing social support is available when needed can improve coping skills, which can reduce the impact of stressful life events, such as living with a chronic health condition [35]. This also relates back to the social health framework [9, 10].

The aim of Element 2: Development of a preliminary synthesis is to provide an initial description of the findings of the included studies. The findings in this review will be presented through textual descriptions, grouping and clustering, and tabulation [33]. In Element 3: Exploration of relationships in the data, the preliminary synthesis is used to get more insight into patterns between the different studies. Exploring the relationships within the data helps to develop an understanding of how and why an intervention or a practice works. The methods that will be used for this review are translation (way to explore relationships across studies) and qualitative case descriptions [33]. Finally, Element 4 aims to assess the robustness and trustworthiness of the synthesis. Where the robustness includes an interpretation of the methodological quality of the included studies, the trustworthiness also includes an assessment of the methods that are used for the synthesis. For this review, the robustness will be addressed with two quality assessment tools to assess the quality of the included studies. The trustworthiness of will be addressed by assessing the strengths and limitations of this review [33].
