# Supplementary material for: Online Peer Support for People With Parkinson Disease: Narrative Synthesis Systematic Review
Source: JMIR Aging. 2022 Jul 27;5(3):e35425. doi: 10.2196/35425 (PMC9377481; doi:10.2196/35425)
Supplement: Multimedia Appendix 2 [file aging_v5i3e35425_app2.docx]

## Multimedia Appendix 2

**Table 3**. CASP checklist for Qualitative Studies

|  | Attard and Coulson [31] | Bakke et al [32] | Davis and Boellstorff ([47] | Stewart Loane et al [43] | Martínez-Pérez et al [48] |
| --- | --- | --- | --- | --- | --- |
| 1 Clear statement of aims? | + | + | + | + | + |
| 2 Qualitative methodology appropriate? | + | + | + | + | + |
| 3 Research design appropriate? | + | + | + | + | + |
| 4 Recruitment strategy appropriate? * | + | + | + | - | + |
| 5 Data collected in a way that addressed the research issue? | + | + | + | + | - |
| 6 Has the relationship between researcher and participant** been adequately considered? | - | - | - | - | - |
| 7 Ethical issues been taken into consideration? | + | - | + | + | - |
| 8 Data analysis sufficiently rigorous? | + | + | + | + | - |
| 9 Clear statement of findings? | + | + | - | + | - |
| 10 How valuable is this research? | + | + | - | + | - |
| Total score | 9 | 8 | 7 | 8 | 4 |

+ = criterion met; – = criterion not met. *In case of qualitative content analysis this item focused on selection of platforms and messages posted. **In case of qualitative content analysis ‘participants’ relates to the data analysis

**Table 4**. Downs & Black checklist for randomised and non-randomised studies

|  | Lieberman et al [46] | Lieberman et al [45] | Lieberman [44] |
| --- | --- | --- | --- |
| 1 Is the hypothesis/aim/ objective clearly described? | 1 | 1 | 1 |
| 2 Are the main outcomes to be measured clearly described in the introduction or methods section? | 1 | 1 | 1 |
| 3 Are the characteristics of the patients clearly described? | 1 | 1 | 1 |
| 4 Are the interventions of interest clearly described? | 1 | 1 | 1 |
| 5 Are the distributions of the principal confounders in each group of subjects to be compared clearly described?** | 0 | 0 | 0 |
| 6 Are the main findings of the study clearly described? | 1 | 1 | 1 |
| 7 Does the study provide estimates of the random variability in the data for the main outcomes? | 1 | 1 | 1 |
| 8 Have all important adverse events that may be a consequence of the intervention been reported? | 0 | 0 | 0 |
| 9 Have the characteristics of patients lost to follow-up been described? | 1 | 1 | 1 |
| 10 Have actual probability values been reported for the main outcomes except where the probability value is less than 0.001? | 1 | 1 | 1 |
| 11 Were the subjects asked to participate in the study representative of the entire population? | 1 | 1 | 0 |
| 12 Were those who were prepared to participate representative of the entire population? | 0 | 0 | 0 |
| 13 Were the staff, places, and facilities where the patients were treated, representative of the treatment the majority of patients receive? | 0 | 0 | 1 |
| 14 Was an attempt made to blind the subjects to the intervention they have received? | N/A | N/A | N/A |
| 15 Was an attempt made to blind those measuring the main outcomes of the intervention? | N/A | N/A | N/A |
| 16 If any of the results of the study were based on “data dredging”, was this made clear? | 1 | 1 | 1 |
| 17 In trials and cohort studies, do the analyses adjust for different lengths of follow-up of patients, or in case of case-control studies, is the time period between the intervention and outcome the same for cases and controls? | 1 | 1 | 1 |
| 18 Were the statistical tests used to assess the main outcomes appropriate? | 1 | 1 | 1 |
| 19 Was compliance with the intervention reliable? | 1 | 1 | 1 |
| 20 Were the main outcome measures used accurate (valid and reliable)? | 1 | 1 | 1 |
| 21 Were the patients in different intervention groups (trials and cohort studies) or were the cases and controls (case-control studies) recruited from the same population? | 1 | 1 | 1 |
| 22 Were study subjects in different intervention groups (trials and cohort studies) or were cases and controls (case-control studies) recruited over the same period of time? | 0 | 0 | 0 |
| 23 Were study subjects randomised to intervention groups? | 0 | 0 | 0 |
| 24 Was the randomised intervention assignment concealed from both patients and healthcare staff until recruitment was complete and irrevocable? | 0 | 0 | 0 |
| 25 Was there adequate adjustment for confounding in the analyses from which the main findings were drawn? | 0 | 0 | 0 |
| 26 Were losses of patients to follow-up taken into account? | 1 | 1 | 1 |
| 27 Did the study have sufficient power to detect a clinically important effect where the probability value for a difference being due to chance is less than 5%? | N/A | N/A | N/A |
| Total score | 16/25  Good | 16/25  Good | 15/25  Fair |

1=criterion met, 0=criterion not met, or unable to determine

*This study is a content analysis of online support groups. Therefore, for this question, ‘patients’ will be the online groups included in this study.

**For this question, a score of 2 indicates criterion met, a score of 1 indicates criterion partially met, and a score of 0 indicates criterion not met.
